# *Vital Signs:* Deaths Among Persons with Diagnosed HIV Infection, United States, 2010–2018

**DOI:** 10.15585/mmwr.mm6946a1

**Published:** 2020-11-20

**Authors:** Karin A. Bosh, Anna Satcher Johnson, Angela L. Hernandez, Joseph Prejean, Jocelyn Taylor, Rachel Wingard, Linda A. Valleroy, H. Irene Hall

**Affiliations:** 1 Division of HIV/AIDS Prevention, National Center for HIV/AIDS, Viral Hepatitis, STD, and TB Prevention, CDC.

## Abstract

**Background:**

Life expectancy for persons with human immunodeficiency virus (HIV) infection who receive recommended treatment can approach that of the general population, yet HIV remains among the 10 leading causes of death among certain populations. Using surveillance data, CDC assessed progress toward reducing deaths among persons with diagnosed HIV (PWDH).

**Methods:**

CDC analyzed National HIV Surveillance System data for persons aged ≥13 years to determine age-adjusted death rates per 1,000 PWDH during 2010–2018. Using the *International Classification of Diseases, Tenth Revision*, deaths with a nonmissing underlying cause were classified as HIV-related or non–HIV-related. Temporal changes in total deaths during 2010−2018 and deaths by cause during 2010–2017 (2018 excluded because of delays in reporting), by demographic characteristics, transmission category, and U.S. Census region of residence at time of death were calculated.

**Results:**

During 2010–2018, rates of death decreased by 36.6% overall (from 19.4 to 12.3 per 1,000 PWDH). During 2010–2017, HIV-related death rates decreased 48.4% (from 9.1 to 4.7), whereas non–HIV-related death rates decreased 8.6% (from 9.3 to 8.5). Rates of HIV-related deaths during 2017 were highest by race/ethnicity among persons of multiple races (7.0) and Black/African American persons (5.6), followed by White persons (3.9) and Hispanic/Latino persons (3.9). The HIV-related death rate was highest in the South (6.0) and lowest in the Northeast (3.2).

**Conclusion:**

Early diagnosis, prompt treatment, and maintaining access to high-quality care and treatment have been successful in reducing HIV-related deaths and remain necessary for continuing reductions in HIV-related deaths.

## Introduction

Persons with human immunodeficiency virus (HIV) infection require lifelong treatment to reduce HIV-related morbidity and mortality; advances in HIV treatment have resulted in a life expectancy that approaches that of the general population (*1*,*2*). Deaths attributable to HIV infection are preventable, yet during 2017, HIV was still among the 10 leading causes of death among certain population groups (*3*).

The National HIV Surveillance System (NHSS) is the primary source of population-based information about HIV in the United States (*4*). A previous analysis demonstrated that, during 1990–2011, deaths among persons with stage 3 HIV infection (acquired immunodeficiency syndrome [AIDS]) decreased, with larger decreases in HIV-attributable deaths (−89%) than in non–HIV-attributable deaths (−57%) (*5*). On the basis of increasing evidence of the benefits of antiretroviral therapy both for persons with HIV and for preventing secondary transmission, treatment guidelines were updated in 2012 to recommend antiretroviral therapy for all persons with HIV (*6*). A national target for reducing the death rate among persons with diagnosed HIV (PWDH) by ≥33% during 2010–2020 was established to encourage progress toward improving health outcomes among PWDH (*7*). Using NHSS data, CDC assessed such progress, with an emphasis on HIV-related deaths, at the national and state levels.

## Methods

CDC analyzed NHSS data reported through December 2019 regarding deaths during 2010–2018 among persons aged ≥13 years with diagnosed HIV infection. Using the *International Classification of Diseases, Tenth Revision* (ICD-10) codes associated with the underlying cause, deaths were classified as HIV-related or non–HIV-related.* Annual deaths (2010–2018) and deaths by cause (2010–2017 because of delays in reporting) were assessed by demographic characteristics, transmission category, and U.S. region of residence at time of death. National-level results include persons with a residence at time of death in the 50 states or the District of Columbia; jurisdiction-level results also include persons with a residence at time of death in Puerto Rico.

Age-adjusted rates per 1,000 PWDH were calculated using the U.S. 2000 standard population. For HIV-related deaths, CDC calculated an absolute and a relative disparity measure for race/ethnicity and assessed change from 2010 to 2017.^†^^,^^§^ For all measures, only stable rates (calculated on the basis of ≥12 deaths) and rates by cause of death for groups among whom ≥85% of deaths had a known cause (i.e., complete cause of death reporting) were assessed for temporal changes and for differences among groups.

## Results

**FIGURE 1:**
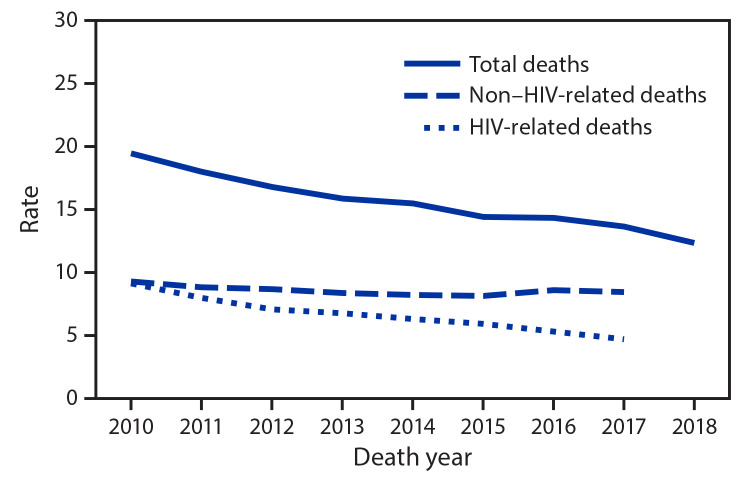
Age-adjusted rates* of total deaths,^†^ human immunodeficiency virus (HIV)–related deaths,^§^ and non–HIV-related deaths among persons aged ≥13 years with diagnosed HIV infection — United States, 2010–2018^¶^ * Rates per 1,000 persons with diagnosed HIV infection. Rates age-adjusted using the U.S. 2000 standard population. ^†^ Deaths among persons with diagnosed HIV infection regardless of cause of death (n = 16,742 in 2010; n = 15,483 in 2018). ^§^ HIV-related deaths include deaths with an underlying cause with an *International Classification of Diseases, Tenth Revision* code of B20-B24, O98.7, or R75. Non–HIV-related deaths include all other deaths with a known underlying cause. ^¶^ Deaths by cause available through 2017 because of reporting delays.

During 2010–2018, the number of deaths among PWDH decreased by 7.5%, from 16,742 during 2010 to 15,483 during 2018; the rate of death decreased by 36.6% overall (Figure 1). The rate of HIV-related deaths decreased 48.4% from 9.1 per 1,000 PWDH during 2010 to 4.7 per 1,000 PWDH during 2017, whereas the rate of non–HIV-related deaths decreased 8.6% from 9.3 in 2010 to 8.5 in 2017 (Figure 1). The rate of HIV-related deaths during 2010–2017 decreased in all regions and for all gender, age, race/ethnicity, and transmission category groups. (Supplementary Table 1, https://stacks.cdc.gov/view/cdc/96933). The absolute rate difference disparity measure for HIV-related deaths between Hispanic/Latino persons and White persons decreased to zero (3.9 per 1,000 PWDH in both populations) in 2017. During 2010–2017, the absolute rate difference disparity measure between Black/African American (Black) persons and White persons decreased by 66.0%, and between persons of multiple races and White persons decreased 36.7%. The relative rate ratio disparity measure between Black persons and White persons decreased 23.2%, between Hispanic/Latino persons and White persons decreased 17.7%, but between persons of multiple races and White persons increased 2.3%.

Rates of HIV-related deaths during 2017 were higher among females (5.4 per 1,000 PWDH) than males (4.5) and transgender females (females assigned male sex at birth) (4.3), and highest among persons of multiple races (7.0) and Black persons (5.6), followed by White persons (3.9) and Hispanic/Latino persons (3.9) (Table 1). The rates of HIV-related deaths increased with age, from 1.6 among PWDH aged 13–24 years to 8.4 among persons aged ≥55 years. However, the proportion of deaths that were HIV-related decreased with increasing age from 48.6% among PWDH aged 13–24 years with a known cause of death to 30.0% among PWDH aged ≥55 years with a known cause of death because the rate of non–HIV-related death increased with age more than the rate of HIV-related death. Among males, the rate of HIV-related death was lower among those whose infection was attributed to male-to-male sexual contact (3.9) than among those whose infection was attributed to other transmission categories; among females, the rate was lower among those with infection attributed to heterosexual contact (4.6) than among those in other transmission categories. The rate of HIV-related deaths was highest in the South (6.0) and lowest in the Northeast (3.2).

**TABLE 1:** Total deaths, human immunodeficiency virus (HIV)–related deaths, and non–HIV-related deaths among persons aged ≥13 years with diagnosed HIV infection, by selected characteristics — United States, 2017

Characteristic	Total		HIV-related*				Non–HIV-related*	
	No.	Age-adjusted rate per 1,000 PWDH^†^	No.	% of deaths related to HIV	% of deaths with known cause related to HIV	Age-adjusted rate per 1,000 PWDH^†^	No.	Age-adjusted rate per 1,000 PWDH^†^
**Gender**								
Male	12,256	13.5	4,033	32.9	34.1	4.5	7,779	8.5
Female	3,994	14.1	1,466	36.7	37.7	5.4	2,425	8.3
Transgender male-to-female^§^	103	14.8	33	32.0	32.4	4.3	69	10.2
Transgender female-to-male^§^	4	18.0	2	50.0	50.0	10.7	2	7.3
Additional gender identity^¶^	1	13.8	0	0	0	0	1	13.8
**Age at death, yrs**								
13–24	151	3.5	70	46.4	48.6	1.6	74	1.7
25–34	1,048	6.5	492	46.9	48.8	3.0	516	3.2
35–44	1,838	9.4	826	44.9	46.4	4.2	954	4.9
45–54	4,470	14.4	1,584	35.4	36.6	5.1	2,740	8.9
≥55	8,851	28.9	2,562	28.9	30.0	8.4	5,992	19.5
**Race/Ethnicity**								
American Indian/Alaska Native	44	13.5	8	18.2	21.1	3.3	30	8.3
Asian**	88	6.3	27	30.7	39.1	1.5	42	3.1
Black/African American	7,197	15.1	2,620	36.4	37.3	5.6	4,412	9.2
Hispanic/Latino^††^	2,694	11.1	955	35.4	37.2	3.9	1,609	6.6
Native Hawaiian/Other Pacific Islander**	9	15.0	0	0	0	0	2	2.5
White	5,255	13.3	1,546	29.4	30.5	3.9	3,520	8.9
Multiple races	1,069	19.5	378	35.4	36.5	7.0	659	12.0
**Transmission category^§§^**								
**Male adult or adolescent^¶¶^**								
Male-to-male sexual contact	7,010	11.4	2,408	34.4	35.6	3.9	4,351	7.1
Injection drug use	2,168	22.7	590	27.2	28.1	6.2	1,506	15.8
Male-to-male sexual contact and injection drug use	1,373	19.1	444	32.4	33.3	6.1	889	12.6
Heterosexual contact***	1,705	16.1	579	34.0	35.6	5.8	1,046	9.5
Other^†††^	104	19.0	44	42.4	43.4	6.6	57	11.9
Subtotal	12,360	13.5	4,066	32.9	34.1	4.5	7,849	8.5
**Female adult or adolescent^¶¶^**								
Injection drug use	1,373	21.6	454	33.0	33.7	7.7	893	13.1
Heterosexual contact***	2,553	12.0	974	38.2	39.3	4.6	1,506	7.1
Other^†††^	72	16.2	40	56.2	59.6	7.0	27	8.9
Subtotal	3,998	14.1	1,468	36.7	37.7	5.4	2,427	8.3
**U.S. Census region of residence at time of death**								
Midwest	1,901	14.1	602	31.7	32.3	4.4	1,263	9.4
Northeast	3,689	12.0	941	25.5	26.8	3.2	2,576	8.2
South	8,040	15.5	3,092	38.5	39.1	6.0	4,822	9.2
West	2,728	11.4	899	33.0	35.8	3.9	1,615	6.6
**Total**	**16,358**	**13.6**	**5,534**	**33.8**	**35.0**	**4.7**	**10,276**	**8.5**

**Abbreviation:** PWDH = persons with diagnosed HIV infection.

* HIV-related deaths include deaths with an underlying cause with an *International Classification of Diseases, Tenth Revision* code of B20–B24, O98.7, or R75. Non–HIV-related deaths include all other deaths with a known underlying cause. Deaths with an unknown underlying cause are excluded.

^†^ PWDH includes persons living with HIV infection at the end of the calendar year plus the number of diagnoses of HIV infection during the current calendar year. Rates age-adjusted using the U.S. 2000 standard population. Rates presented by age at time of death are not age-adjusted. Rates and percentage change calculated on the basis of <12 deaths are considered unstable and should be interpreted with caution.

^§^ “Transgender male-to-female” includes persons who were assigned “male” sex at birth but have ever identified as “female.” “Transgender female-to-male” includes persons who were assigned “female” sex at birth but have ever identified as “male.”

^¶^ Additional gender identity examples include “bigender,” “gender queer,” and “two-spirit.”

** Data by cause of death should be interpreted with caution because <85% of reported deaths were reported with a known underlying cause of death.

^††^ Hispanic/Latino persons can be of any race.

^§§^ Data have been statistically adjusted to account for missing transmission category; therefore, values might not sum to column subtotals and total.

^¶¶^ Data presented are based on sex at birth and include transgender persons.

*** Heterosexual contact with a person known to have, or to be at high risk for, HIV infection.

^†††^ Includes hemophilia, blood transfusion, perinatal, and risk factor not reported or not identified.

**FIGURE 2:**
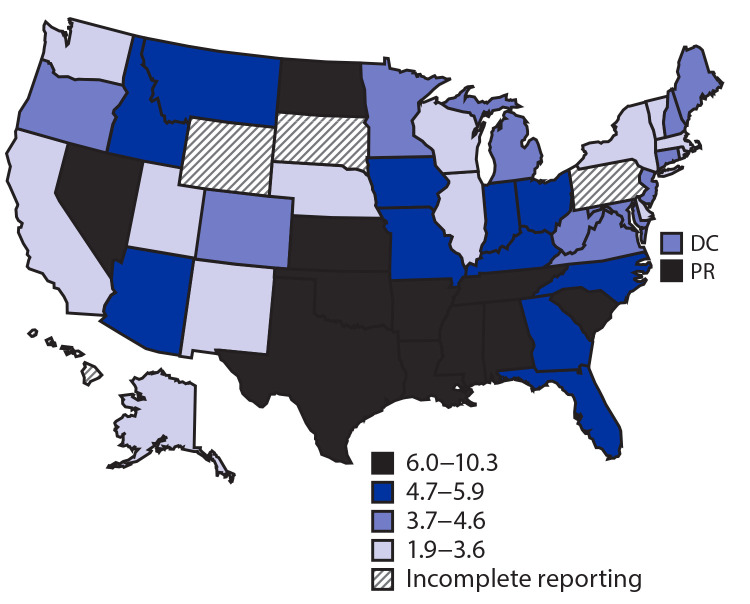
Age-adjusted rates* of human immunodeficiency virus (HIV)–related deaths among persons aged ≥13 years with diagnosed HIV infection, by area of residence at time of death — United States and Puerto Rico, 2017 **Abbreviations:** DC = District of Columbia; PR = Puerto Rico. * Rates per 1,000 persons with diagnosed HIV infection. Rates age-adjusted using the U.S. 2000 standard population. HIV-related deaths include deaths with an underlying cause with an *International Classification of Diseases, Tenth Revision,* code of B20 –B24, 098.7, or R75. Other U.S. dependent areas are excluded because they do not report underlying cause of death information. Jurisdictions with striped shading are those with <85% of deaths in 2017 with a known underlying cause of death. Rates from Alaska, Idaho, Maine, Montana, Nebraska, New Hampshire, New Mexico, North Dakota, Rhode Island, Utah, Vermont, and West Virginia are calculated based on <12 deaths and should be interpreted with caution.

In all areas with complete cause-of-death reporting and with stable rates, HIV-related deaths were lower during 2017 than in 2010 (Supplementary Table 2, https://stacks.cdc.gov/view/cdc/96934). Rates of HIV-related deaths during 2017 varied by jurisdiction; rates were highest in Mississippi (10.3 per 1,000 PWDH), Puerto Rico (9.2), and South Carolina (8.0), and lowest in New York (3.0), Massachusetts (3.1) and Delaware (3.2) (Figure 2). During 2017, rates of HIV-related deaths by race/ethnicity varied by jurisdiction (Table 2) (Supplementary Table 3, https://stacks.cdc.gov/view/cdc/96934). Rates of HIV-related death were highest among White persons in South Carolina (10.1), Oklahoma (7.5), and Arkansas (6.5); highest among Black persons in Mississippi (11.5), Louisiana (8.8), South Carolina (8.2), and Nevada (8.2); and highest among Hispanic/Latino persons in Puerto Rico (9.2), Texas (6.5), and Arizona (6.2).

**TABLE 2:** Total deaths and human immunodeficiency virus (HIV)–related deaths among persons aged ≥13 years with diagnosed HIV infection, by area of residence at time of death, and selected race/ethnicity categories — United States and Puerto Rico,* 2017

Area of residence	All races/ethnicities			Black/African American				Hispanic/Latino				White			
	Total			Total		HIV-related^†^		Total		HIV-related^†^		Total		HIV-related^†^	
	No.	Age-adjusted rate per 1,000 PWDH^§^	% of deaths with a known cause	No.	Age-adjusted rate per 1,000 PWDH^§^	No.	Age-adjusted rate per 1,000 PWDH^§^	No.	Age-adjusted rate per 1,000 PWDH^§^	No.	Age-adjusted rate per 1,000 PWDH^§^	No.	Age-adjusted rate per 1,000 PWDH^§^	No.	Age-adjusted rate per 1,000 PWDH^§^
Alabama	257	17.8	98.4	150	17.5	60	6.5	5	11.4	2	5.2	83	19.5	29	6.1
Alaska	7	8.5	100.0	0	0	0	0	1	11.8	0	0	5	14.0	1	1.6
Arizona	247	13.5	99.6	29	14.9	10	6.1	56	12.3	26	6.2	145	14.7	48	4.6
Arkansas	107	17.0	99.1	33	12.3	16	6.2	6	15.7	3	7.9	61	19.2	21	6.5
California	1,717	11.0	89.7	327	12.1	118	4.4	509	10.2	195	3.9	724	10.4	193	2.8
Colorado	132	8.4	100.0	13	5.9	6	2.8	33	12.2	12	4.9	77	7.2	33	3.1
Connecticut	198	15.0	99.5	72	14.7	18	4.9	57	12.6	15	3.7	64	18.8	16	4.0
Delaware	67	13.8	100.0	50	18.0	13	4.6	4	13.6	1	3.8	11	6.5	1	0.5
District of Columbia	237	13.4	97.0	202	16.2	59	5.3	3	3.5	1	1.2	14	4.6	2	0.6
Florida	2,122	15.6	98.4	1,049	17.5	424	7.2	329	11.0	124	4.1	683	16.2	199	4.7
Georgia	823	14.5	97.7	559	15.3	244	6.5	43	12.5	18	4.2	158	12.0	38	2.9
Hawaii^¶^	37	15.2	35.1	1	5.6	0	0	1	3.3	1	3.3	12	7.0	2	0.8
Idaho	20	13.6	100.0	0	0	0	0	1	2.6	1	2.6	17	16.4	6	5.8
Illinois	498	12.8	99.2	289	16.6	76	4.4	48	7.3	14	1.8	124	11.2	30	2.1
Indiana	212	17.1	97.2	66	15.7	20	5.4	10	10.6	6	6.4	126	20.3	44	5.6
Iowa	44	13.1	100.0	11	16.9	6	9.7	3	7.0	1	2.3	26	11.3	10	4.4
Kansas	47	14.4	97.9	11	16.0	7	11.1	6	10.8	2	2.1	29	14.1	12	5.1
Kentucky	138	16.4	97.8	44	17.0	17	6.3	5	6.4	3	3.9	82	16.8	24	4.8
Louisiana	411	18.5	98.8	276	19.0	125	8.8	7	5.8	1	0.7	115	19.8	34	6.3
Maine	35	15.6	100.0	1	2.5	0	0	2	12.0	1	4.9	31	19.0	7	5.9
Maryland	597	14.7	98.3	412	14.0	132	4.4	26	9.5	9	3.0	84	18.9	19	3.8
Massachusetts	307	11.6	96.1	64	8.3	14	2.6	90	13.1	27	4.2	144	13.7	33	2.8
Michigan	273	15.6	99.3	160	17.2	49	5.1	13	14.9	4	3.2	88	12.5	27	3.8
Minnesota	91	9.3	98.9	27	8.3	15	4.0	6	6.9	3	2.1	52	10.2	19	4.2
Mississippi	220	21.2	98.6	158	22.1	85	11.5	9	36.7	6	21.3	44	19.3	12	5.5
Missouri	213	14.5	97.2	97	16.7	38	6.5	10	13.1	6	7.8	95	13.6	30	3.9
Montana	15	20.9	100.0	0	0	0	0	4	93.8	2	34.1	8	11.4	1	1.6
Nebraska	33	14.3	93.9	11	20.2	4	5.7	2	6.9	1	5.4	20	14.9	4	2.5
Nevada	174	16.1	97.7	50	20.5	19	8.2	23	9.6	9	3.4	90	15.9	26	4.6
New Hampshire	21	13.7	95.2	0	0	0	0	3	15.5	0	0	18	17.2	6	6.0
New Jersey	646	13.8	98.6	324	15.1	105	5.2	141	12.1	43	3.9	118	11.9	25	3.0
New Mexico	61	13.1	98.4	2	8.4	0	0	24	11.2	4	2.0	25	12.4	3	1.4
New York	1,787	10.7	98.3	661	10.7	164	3.0	635	11.0	170	3.0	247	7.2	59	1.9
North Carolina	547	15.1	98.9	338	15.3	137	6.1	19	7.9	8	3.5	152	15.1	48	4.6
North Dakota	3	8.9	100.0	0	0	0	0	1	131.2	1	131.2	2	8.7	1	4.4
Ohio	377	15.5	97.9	152	15.2	53	5.5	21	10.7	7	3.7	176	16.7	50	4.2
Oklahoma	109	16.8	95.4	22	13.5	11	7.1	6	8.9	1	1.0	65	20.3	22	7.5
Oregon	115	12.4	100.0	5	9.6	1	1.9	9	7.9	6	5.3	96	13.4	36	5.1
Pennsylvania^¶^	650	14.5	81.8	301	14.4	55	2.5	105	14.7	21	3.0	202	14.4	34	2.4
Puerto Rico	401	19.1	99.0	0	0	0	0	400	19.1	181	9.2	1	21.9	0	0
Rhode Island	33	9.6	97.0	7	8.6	3	3.5	6	8.6	2	3.2	17	9.5	4	2.2
South Carolina	337	17.2	97.9	236	17.7	110	8.2	5	6.4	2	2.3	83	19.3	34	10.1
South Dakota^¶^	9	12.4	33.3	2	9.1	1	3.9	0	0	0	0	4	10.0	0	0
Tennessee	332	18.2	99.4	189	19.9	81	7.7	6	9.2	2	3.2	128	17.3	48	5.8
Texas	1,413	15.1	98.9	557	17.3	245	7.4	359	12.8	182	6.5	409	14.6	157	5.8
Utah	22	8.4	95.5	1	4.2	1	4.2	3	9.8	0	0	16	7.0	8	3.4
Vermont	12	11.4	100.0	0	0	0	0	1	7.5	0	0	10	12.2	3	3.2
Virginia	292	10.5	98.3	180	11.8	60	4.1	9	3.3	6	2.1	79	9.0	27	3.2
Washington	175	10.4	97.1	24	10.8	9	4.2	19	9.3	6	2.8	112	10.8	35	3.8
West Virginia	31	13.8	100.0	4	7.5	1	2.1	1	11.9	0	0	24	15.8	6	4.6
Wisconsin	101	12.7	100.0	30	11.7	8	3.3	9	9.0	0	0	57	13.8	18	4.2
Wyoming^¶^	6	27.2	66.7	0	0	0	0	0	0	0	0	3	9.5	1	3.5

**Abbreviation:** PWDH = persons with diagnosed HIV infection.

* Other U.S. dependent areas are excluded because they do not report underlying cause of death information.

^†^ HIV-related deaths include deaths with an underlying cause with an *International Classification of Diseases, Tenth Revision* code of B20–B24, O98.7, or R75. Non–HIV-related deaths include all other deaths with a known underlying cause. Deaths with an unknown underlying cause are excluded.

^§^ PWDH includes persons living with HIV infection at the end of the calendar year plus the number of diagnoses of HIV infection during the current calendar year. Rates age-adjusted using the U.S. 2000 standard population. Rates calculated based on consideration that analyses of data with <12 deaths are considered unstable and should be interpreted with caution.

^¶^ Proportion of deaths with a known underlying cause of death is <85%.

## Discussion

By 2018, the rate of death among PWDH in the United States had decreased by 36.6% from what it was in 2010, surpassing the 2020 national target of ≥33% (*7*). This decrease, which was primarily attributable to reductions in HIV-related deaths, likely reflects the increase during 2010–2018 in the proportion of persons who knew their serostatus from 82.2% to 86.2% and the implementation of updated treatment guidelines resulting in increased viral suppression among PWDH from 46.0% to 64.7% (*6*,*8*). Absolute and relative differences in HIV-related deaths among Black persons and Hispanic/Latino persons, compared with those among White persons, also decreased during 2010–2017. This reduction likely reflects a greater relative improvement during 2012–2017 in the time from diagnosis to viral suppression among Black persons, compared with White persons (*9*), and reduced disparities during 2010–2016 in viral suppression among Black persons and Hispanic/Latino persons, compared with White persons (*10*). These findings highlight how successes in identifying HIV infections, initiating treatment, and achieving viral suppression among PWDH improve health outcomes.

Despite success in reducing rates of HIV-related deaths among PWDH, differences still exist by gender, race/ethnicity, age, transmission category, and region. Variation in timely diagnosis and treatment initiation, along with ongoing treatment, likely contributes to differences in HIV-related deaths. During 2015, delays in HIV diagnosis were longer among non-White racial/ethnic groups and males with HIV infection attributed to heterosexual contact (*11*). Timely initiation of treatment, as measured by the proportion of persons with suppressed viral loads ≤6 months after diagnosis, and receipt of ongoing, recommended treatment, as measured by the proportion of PWDH with a suppressed viral load, varied during 2017 by gender, age, race/ethnicity, transmission category, and region (*8*,*12*); populations with higher rates of HIV-related deaths were less likely to have evidence of timely initiation of treatment and ongoing treatment as demonstrated through lower proportions of viral suppression in the population.

Prevalence of HIV infection and the number of HIV-related deaths were greatest by race/ethnicity among Black persons and by U.S. region in the South (*4*). Rates of HIV-related deaths were also high among these two populations. Higher levels of poverty, unemployment, and persons uninsured, challenges associated with accessing care, and HIV-related stigma likely affect timely diagnosis and access to treatment and contribute to higher rates of HIV-related deaths (*13*,*14*). Expanded efforts to address these and other structural barriers are critical to improving health outcomes, including reducing differences in HIV-related death rates, especially among Black persons and persons in the South.

Although rates of HIV-related deaths were lower among younger PWDH, the proportion of HIV-related deaths among younger PWDH (ages 13–44 years) was higher than that among older PWDH; this is concerning because HIV-related deaths are preventable. Higher proportions of undiagnosed HIV infections and lower levels of viral suppression are more common among younger persons (*8*,*15*). Additional efforts are needed to ensure younger persons are aware of their infection and able to access and adhere to recommended, ongoing HIV treatment to improve health outcomes.

CDC supports numerous activities for identifying HIV infections: initiating treatment as quickly as possible and ensuring ongoing treatment; addressing social barriers to HIV prevention and treatment efforts; and expanding opportunities for persons to test for HIV infection and receive the results on their own (i.e., self-testing), which allows persons who might not otherwise take a test to learn their HIV status (*16*). CDC’s Integrated HIV Surveillance and Prevention Programs for Health Departments, initiated in 2018, includes critical activities to enable state and local health departments to improve identification of HIV infections and increase viral suppression among PWDH (*17*). CDC’s national campaign, Let’s Stop HIV Together, supports efforts to end HIV stigma and promote HIV testing, prevention, and treatment (*18*). Ending the HIV Epidemic: A Plan for America is an initiative for reducing HIV infections in the United States by ≥90% by 2030; it focuses on strategies regarding diagnosis, treatment, prevention, and response to HIV infection in communities most affected by HIV (*19*). In addition to decreasing the risk for ongoing HIV transmission, prompt diagnosis and improving timely and continuing access to HIV treatment should also improve health outcomes for PWDH and prevent HIV-related deaths.

The findings in this report are subject to at least two limitations. First, cause-of-death information on death certificates is typically completed by funeral directors, attending physicians, medical examiners, or coroners (*3*). HIV-related deaths might be underreported because of lack of knowledge about the correct documentation needed or reluctance to include HIV on the death certificate because of possible stigma (*5*). An assessment of Florida’s HIV surveillance data for 2000–2011 indicated that HIV-related deaths were underestimated in the surveillance system by approximately 9% (*20*). Second, the proportion of deaths with a known cause was <100%. Overall, the proportion of deaths with a known cause was high for the United States (94.6% in 2010 and 96.7% in 2017); however, the proportion of deaths with a known cause was lower for certain demographic groups (e.g., Asian persons) and for certain jurisdictions (e.g., Hawaii during 2017).

Deaths among persons with HIV have decreased, and by 2018 had surpassed the 2020 national target, primarily because of a reduction in HIV-related deaths. Deaths caused by HIV infection have likely decreased because of improvements in diagnosing infections and in treatment and medical care. However, differences in HIV-related death rates still exist for multiple populations. Diagnosing HIV infection early, treating it promptly, and maintaining access to high-quality care and treatment over a lifetime can improve life expectancy and reduce differences in rates of deaths across all populations.

**Summary**

**What is already known about this topic?**

HIV remains among the 10 leading causes of death among certain populations, although deaths attributable to HIV infection are preventable.

**What is added by this report?**

Deaths among persons with diagnosed HIV (PWDH) decreased, primarily because of decreases in HIV-related deaths. The age-adjusted rate per 1,000 PWDH of HIV-related deaths decreased 48% and non–HIV-related deaths decreased 9% during 2010–2017. Differences in HIV-related deaths persist for certain populations.

**What are the implications for public health practice?**

Continued efforts in diagnosing HIV early, promptly initiating treatment, and maintaining access to high-quality care and treatment are necessary for continuing progress in reducing deaths and eliminating differences across populations.

## Footnotes

* HIV-related: deaths with an ICD-10 code of B20–B24, 098.7, or R75 for the underlying cause; non–HIV-related: all other deaths with a nonmissing ICD-10 code for the underlying cause.

† Absolute rate difference disparity measure: Difference between age-adjusted rate per 1,000 PWDH among selected race/ethnicity and White persons (population with lowest rate during 2010 among those with rates where ≥85% of deaths had a known cause). Change in the absolute rate difference disparity measure during 2010–2017 was calculated as ([absolute disparity measure in 2017 – absolute disparity measure in 2010]/absolute disparity measure in 2010) x 100.

§ Relative rate ratio disparity measure: Ratio of age-adjusted rates per 1,000 PWDH for selected race/ethnicity, compared with White persons (population with lowest rate during 2010 among those with rates where ≥85% of deaths had a known cause). Change in the relative rate ratio disparity measure during 2010–2017 was calculated as ([relative disparity measure in 2017 – relative disparity measure in 2010]/relative disparity measure in 2010) x 100.
